# Estimating the clinical cost of drug development for orphan versus non-orphan drugs

**DOI:** 10.1186/s13023-018-0990-4

**Published:** 2019-01-10

**Authors:** Kavisha Jayasundara, Aidan Hollis, Murray Krahn, Muhammad Mamdani, Jeffrey S. Hoch, Paul Grootendorst

**Affiliations:** 1 Leslie Dan Faculty of Pharmacy, University of Toronto, Toronto, Canada; 2 Department of Economics, University of Calgary, Calgary, Canada; 3 Toronto Health Economics and Technology Assessment Collaborative, Toronto, Canada; 4 Li Ka Shing Knowledge Institute, St. Michael’s Hospital, Toronto, Canada; 5 Institute of Health Policy, Management and Evaluation, University of Toronto, Toronto, Canada; 6 University of California, Davis, USA

**Keywords:** Cost of drug development, Orphan drugs, Rare diseases

## Abstract

**Background:**

High orphan drug prices have gained the attention of payers and policy makers. These prices may reflect the need to recoup the cost of drug development from a small patient pool. However, estimates of the cost of orphan drug development are sparse.

**Methods:**

Using publicly available data, we estimated the differences in trial characteristics and clinical development costs with 100 orphan and 100 non-orphan drugs.

**Results:**

We found that the out-of-pocket clinical costs per approved orphan drug to be $166 million and $291 million (2013 USD) per non-orphan drug. The capitalized clinical costs per approved orphan drug and non-orphan drug were estimated to be $291 million and $412 million respectively. When focusing on new molecular entities only, we found that the capitalized clinical cost per approved orphan drug was half that of a non-orphan drug.

**Conclusions:**

More discussion is needed to better align on which cost components should be included in research and development costs for pharmaceuticals.

**Electronic supplementary material:**

The online version of this article (10.1186/s13023-018-0990-4) contains supplementary material, which is available to authorized users.

## Background

In recent years, pharmaceutical companies have increasingly focused on developing drugs for rare diseases [1]. This is partly due to technical advances that facilitate the identification of the genetic causes of rare diseases [2]. Country level initiatives and incentives for the development of drugs that treat rare diseases, i.e. orphan drugs, have also stimulated research efforts in this area. Since the launch of these initiatives, more than 400 orphan drugs have been approved [1]. Orphan drugs have large revenue generating potential, in part because of their high prices [3]. The global orphan drug market is estimated to reach US $209 billion by 2022 accounting for 21.4% of total branded prescription drug sales [4].

As the number of approved orphan drugs increased, the high prices associated with some of them have become the topic of much debate. Imiglucerase, an enzyme replacement therapy to treat Gaucher’s disease, might cost as much as $400 000 USD per year for an adult patient [5]. A drug that treats paroxysmal nocturnal hemoglobinuria, eculizumab, can cost up to US $500,000 per patient per year [6]. Kalydeco, used to treat a subpopulation of cystic fibrosis patients, exceeds $300,000 USD per year per patient [7]. Some believe that pharmaceutical companies price drugs monopolistically, protected by patent rights, to maximize their profits [8, 9]. Others believe that the high prices for orphan drugs simply allow drug research and development (R&D) and production costs to be recouped from a relatively small patient pool. Thus, according to this perspective, drug prices should be regulated, at least in part, on the basis of R&D and production costs.

For those interested in engaging in cost-based pricing, evidence on R&D costs is essential. Moreover, knowledge of the cost of drug development is useful when assessing public policies aimed to stimulate drug development and foster innovation [10]. However, to our knowledge, there are no estimates in the literature of the cost of development of drugs that treat rare diseases. DiMasi et al estimated the out-of-pocket cost per approved new compound developed in house by US pharmaceutical companies to be $1.4 billion and the capitalized costs to the point of marketing approval to be $2.6 billion (2013 USD) [11]. These estimates were derived from the results of confidential surveys of 10 US pharmaceutical companies and includes both clinical and pre-clinical costs. A more recent study by Prasad et al has estimated the cost of developing a cancer drug to be $648 million (out-of-pocket) and $757 million (capitalized) in 2017 USD dollars [12]. These results were based on an analysis of US Securities and Exchange Commission filings for 10 drug companies. The three-fold difference in these R&D cost estimates complicates cost-based pricing discussions.

In this study, we aim to identify the differences in clinical trial characteristics between orphan and non-orphan drugs using publicly available data and use that information to estimate the clinical R&D costs for both orphan and non-orphan groups. As much as updating estimates of cost of drug development using transparent and reproducible methods is important, our main objective was to better understand the difference between these costs for orphan and non-orphan drugs.

## Methods

A detailed version of the methods can be found in the Additional file 1. We have summarized our methods below.

We randomly selected one hundred orphan drugs approved by the United States Food and Drug Administration (FDA) between Jan 2000 – Dec 2015 using the FDA Orphan Drug Database [13]. Similarly, we randomly selected one hundred non –orphan drugs approved in the same time period using the Drugs@FDA database [14]. FDA approvals for a new indication of an already marketed drug were included in our analysis. Approvals related to new formulations, new manufacturers or new dosage forms were excluded.

For both the orphan and non-orphan drugs identified, clinicaltrials.gov was searched for all related clinical trials for the approved indication. These data allowed us to record the number of trials, number of subjects enrolled per trial, and trial duration, by trial phase. We categorized phase 1/2 trials as phase 1, phase 2/3 as phase 2 and bioequivalence studies as phase 1. Trials that did not report on trial phase were randomly allocated into phase 1-3 in the same proportions as studies with this information. Trials with a start date after the FDA approval date and phase 4 trials were excluded. Data on the source of funding and the age and sex of trial subjects was also obtained from the database. In addition, the product monographs and the statistical and medical reviews associated with the New Drug application (NDA) were searched for trial information using the Drugs@FDA website. We created a “restricted” dataset that only included information from the product monographs and FDA review reports. The “All data” dataset included trials that were obtained from clinicaltrials.gov for the same indication but may not have been used for the approval of the drug in question.

Next, we calculated the average cost per trial, by trial phase, for both groups of drugs. This was the product of the average number patients enrolled and the average cost per patient, again stratified by phase and drug group. For non-orphan drugs, the average clinical trial site-based costs per patient by trial phase was obtained through a publicly available data source in 2013 USD [15]. These per patient costs include costs related to investigator and site (institutional overhead, ethics review), recruitment costs, screening, general trial procedures, materials (drug supply, comparator drugs), efficacy assessments (MRIs, CT scans), laboratory procedures (local lab fees, shipping of samples), site-based data management and any site-specific contract research organization costs. We utilized data from a report by EvaluatePharma to calculate the per patient cost for phase 3 trial for orphan drug trials and non-orphan drug trials [16]. These estimates resulted in a ratio of per patient costs for orphan: non-orphan of 2.5:1. Given the similarities in phase 2 and 3 study designs, we assumed that the same ratio for phase 2 trials. The per patient costs for phase 1 trials were assumed to be the same for both orphan and non-orphan groups.

Using the methods used by DiMasi et al [11], we then calculated the expected trial costs for each group based on the average phase-specific trial costs and phase transition probabilities. The transition probabilities for both groups were obtained from Hay et al [17] who reported on probabilities for both orphan drugs and all drugs. In the Hay et al analysis, the proportion of orphan drugs varied from 6% to 15% depending on the clinical stage of development. For the purpose of our analysis, we used the transition probabilities for all drugs reported by Hay et al for the non-orphan group.

These expected costs and overall probability of success for orphan and non-orphan drugs were then used to calculate the expected out-of-pocket costs per approved drug for the two groups in 2013 USD. Costs were then capitalized at 10.5% per annum over the duration of the clinical phase testing and regulatory approval period. This 10.5% discount rate was used by DiMasi et al in their analysis of drug development costs over the period 1994 to 2010 and is estimated using a variant of the capital asset pricing model. All costs were calculated in 2013 USD.

As a scenario analysis, we identified the New Molecular Entities (NMEs) for both orphan and non-orphan groups in our dataset and calculated the cost of clinical drug development specific to these groups. We used the FDA definition of NMEs and relied on the NDA filing data and approval dates to identify the NMEs for each group. In order to estimate the range of drug development costs, various one-way analyses were conducted based on alternative sources of data inputs for number of trials per approved drug, average number of subjects enrolled per trial, average per patient cost by trial phase, trial duration, transition probabilities and discount rate.

## Results

### Clinical Trial Characteristics

Our dataset included 1163 trials with *n* = 561 for non-orphan drugs and *n* = 602 for orphan drugs. The restricted dataset (trials from review reports and product monographs only) included 752 trials with *n* = 410 for non-orphan drugs and *n* = 342 for orphan drugs. Trials that did not report on the trial phase were categorized as ‘missing’. The number of trials and trial characteristics for each group can be seen in Table 1.

**Table 1:** Number of trials and trial characteristics for all data

	Non-Orphan					Orphan				
	Phase					Phase				
	Missing	1	2	3	Total	Missing	1	2	3	Total
Number of trials	57	95	94	315	561	123	122	204	153	602
Proportion (%)	10.16	16.93	16.76	56.15	100.00	20.43	20.27	33.89	25.42	100.00
Sex										
Missing	56	38	37	126	257	111	37	57	24	229
Both	1	40	53	175	269	11	81	134	123	349
Female	0	3	3	13	19	0	2	11	6	19
Male	0	14	1	1	16	1	2	2	0	5
Age										
Missing	56	38	37	126	257	111	37	57	24	229
Adult	0	44	15	26	85	2	8	8	0	18
Adult | Senior	0	11	37	136	184	4	63	93	91	251
Child	0	1	2	4	7	1	1	2	6	10
Child | Adult	0	0	0	1	1	0	5	8	4	17
Child | Adult | Senior	1	1	3	22	27	5	8	36	28	77
Funder Type										
Missing	56	38	37	126	257	111	37	57	24	229
Industry	0	52	55	181	288	3	53	87	106	249
Industry | NIH	0	0	0	0	0	0	0	1	1	2
Industry | NIH | Other	0	1	0	0	1	0	1	3	0	4
Industry | Other	0	3	0	2	5	0	4	18	4	26
NIH	0	1	0	4	5	0	10	14	3	27
NIH |Other	0	0	0	0	0	4	11	8	3	26
NIH | U.S.Fed	0	0	0	0	0	0	0	0	1	1
Other	1	0	2	2	5	5	6	16	11	38
Study Start										
Missing	55	40	42	123	260	40	40	42	28	150
1981 – 1985	0	0	1	0	1	3	0	0	0	3
1986 – 1990	0	0	0	3	3	7	0	3	1	11
1991 - 1995	0	0	1	1	2	30	3	5	9	47
1996 - 2000	1	1	4	25	31	21	8	26	15	70
2001 - 2005	0	24	17	73	114	18	27	77	60	182
2006 - 2010	1	21	15	55	92	1	28	28	26	83
2011 - 2015	0	9	14	35	58	3	16	23	14	56

Note: All values reflect number of trials unless otherwise specified. NIH = National Institutes of Health

The total number of trials for the non-orphan and orphan groups were similar. The majority of the trials for the non-orphan group were in Phase 3 (56%). For the orphan group, the majority of the trials were in Phase 2 (34% for phase 2 and 25% for phase 3). There were also more trials with missing phase information in the orphan group versus the non-orphan group (20% versus 10%). For the trials that reported on the sex of the participants, the majority of the both orphan and non-orphan groups included both sexes. For trials that reported on the age groups of participants, most included adults and seniors for both groups. Trials for the orphan drug group included more children than the non-orphan drugs (17% for orphan, 6% for non-orphan). Most trials were funded by industry for both groups and there were more trials funded by other sources and in combination with NIH for the orphan group. The majority of the trials started between 2001 to 2005 for both orphan and non-orphan groups.

**Fig. 1:**
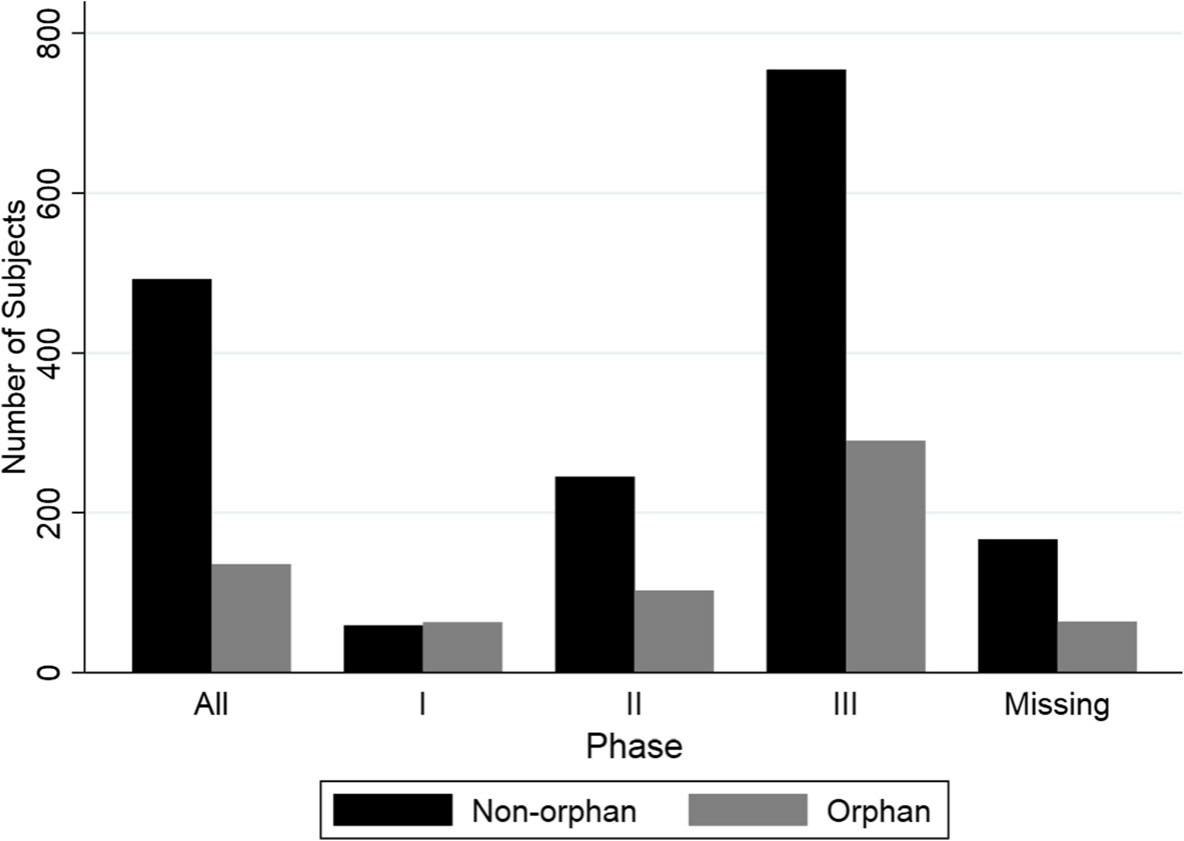
Mean number of subjects in each trial phase for non-orphan and orphan groups**.** Missing = trial phase information not available

The mean number of subjects enrolled in each trial type for all data can be seen in Fig. 1. The number of subjects increased with study phase for both non-orphan and orphan groups. The mean number of subjects for orphan drug groups was less than that of the non-orphan group for phase 2 and 3. The number of subjects in phase 1 appeared to be similar for both orphan and non-orphan groups. The mean numbers for each group by phase can be found in Additional file 2.

**Fig. 2:**
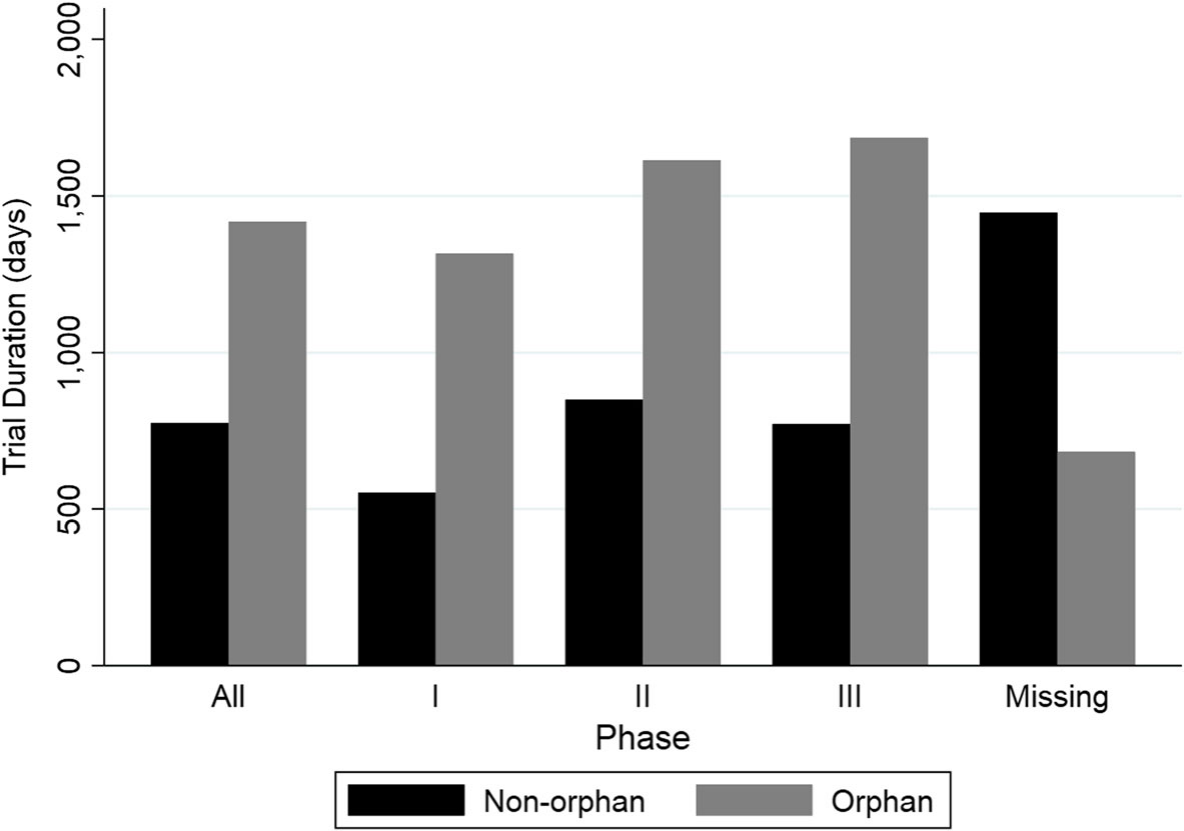
Study Duration for each trial phase for non-orphan and orphan groups. Missing = trial phase information not available

The length of each type of trial in days can be seen in Fig. 2 for the entire dataset. Study end represents the final day in which data was collected (last subject, last visit) while study start represents the day study enrollment started. The length of trials for the orphan drug group was longer than that of the non-orphan group for all phases. The average study duration for orphan drugs was twice that of the non-orphan group (1417 days versus 774 days – see Additional file 2).

### Out-of-pocket Costs

The estimated out-of-pocket clinical costs for each group can be seen in Table 2. For both groups, the out-of-pocket costs increase with phase. The out-of-pocket costs for the non-orphan group were lower for phase 1 and 2 but higher for phase 3 compared to the orphan group. When these costs were translated to expected out-of-pocket costs, the total (for all phases) was higher for the orphan drug group ($30 million for non-orphan versus $55 million for orphan). Using the overall probability of clinical success, the out-of-pocket clinical costs per approved drug was estimated to be $291 million for the non-orphan group and $166 million for the orphan group. The out-of-pocket cost per approved orphan drug was approximately 0.57 times the out-of-pocket cost per non-orphan drug.

**Table 2:** Estimated clinical costs, expected costs and out-of-pocket clinical costs per approved drug

Drug Type	Phase	Estimated out-of-pocket clinical costs (in millions of 2013 USD)	Probability of entering phase	Expected out-of-pocket clinical costs (in millions of 2013 USD)	Overall Probability of clinical success	Out-of-pocket clinical cost per approved drug (in millions of 2013 USD)
Non-orphan	1	$2.6	100%	$2.6	10.44%	$291.4
	2	$9.9	64.5%	$6.4		
	3	$102.7	20.9%	$21.5		
	Total			$30.5		
Orphan	1	$3.8	100%	$3.8	32.93%	$166.1
	2	$23.7	86.8%	$20.6		
	3	$49.9	60.8%	$30.3		
	Total			$54.7		

Estimated out-of-pocket clinical costs = costs accrued by the researcher to conduct the trial, Expected out-of-pocket clinical costs = cost accrued by the researcher adjusted for trial success, Overall probability of success = probability of success from phase 1 to regulatory approval

### Capitalized Costs

Utilizing the out-of-pocket costs, trial durations, regulatory review time and discount rate, the capitalized expected clinical costs and capitalized clinical cost per approved drug were calculated for each group (Table 3). The total capitalized expected costs for the orphan group was higher than that of the non-orphan group ($96 million for orphan versus $43 million for non-orphan). When the overall probability of success was applied to the expected costs to estimate the capitalized cost per approved drug, the estimates were $412 million for the non-orphan group and $291 million for the orphan group. The capitalized cost per approved orphan drug was approximately 0.71 times than that of a non-orphan drug.

**Table 3:** Capitalized expected costs and capitalized cost per approved drug

Drug Type	Phase	Mean phase length (days)	Capitalized expected clinical costs (in millions of 2013 USD)	Capitalized clinical cost per approved drug (in millions of 2013 USD)
Non-orphan	1	624	$4.6	$412.4
	2	849	$10.0	
	3	771	$28.5	
	Total		$43.1	
Orphan	1	1198	$8.8	$291.3
	2	1463	$40.5	
	3	1506	$46.6	
	Total		$95.9	

### New Molecular Entities Only

**Fig. 3:**
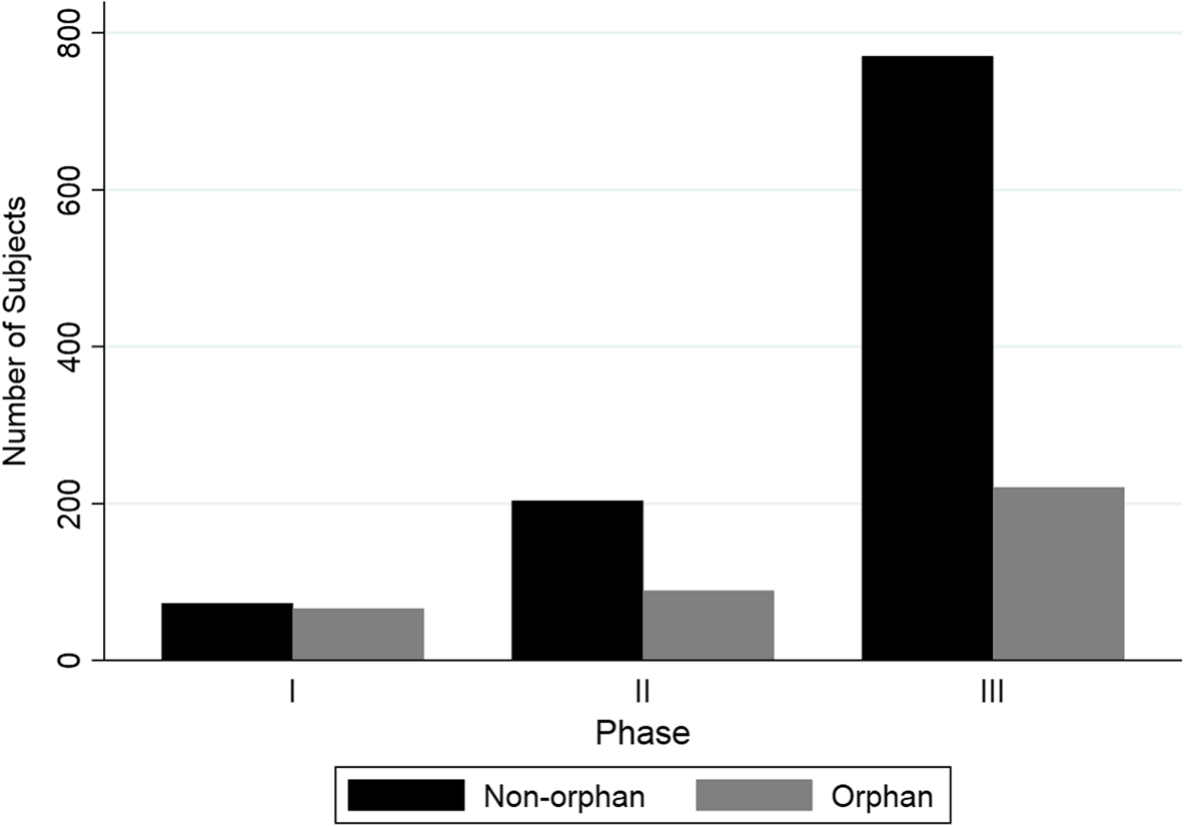
Number of subjects by trial phase for NMEs only. NMEs = New Molecular Entities

**Fig. 4:**
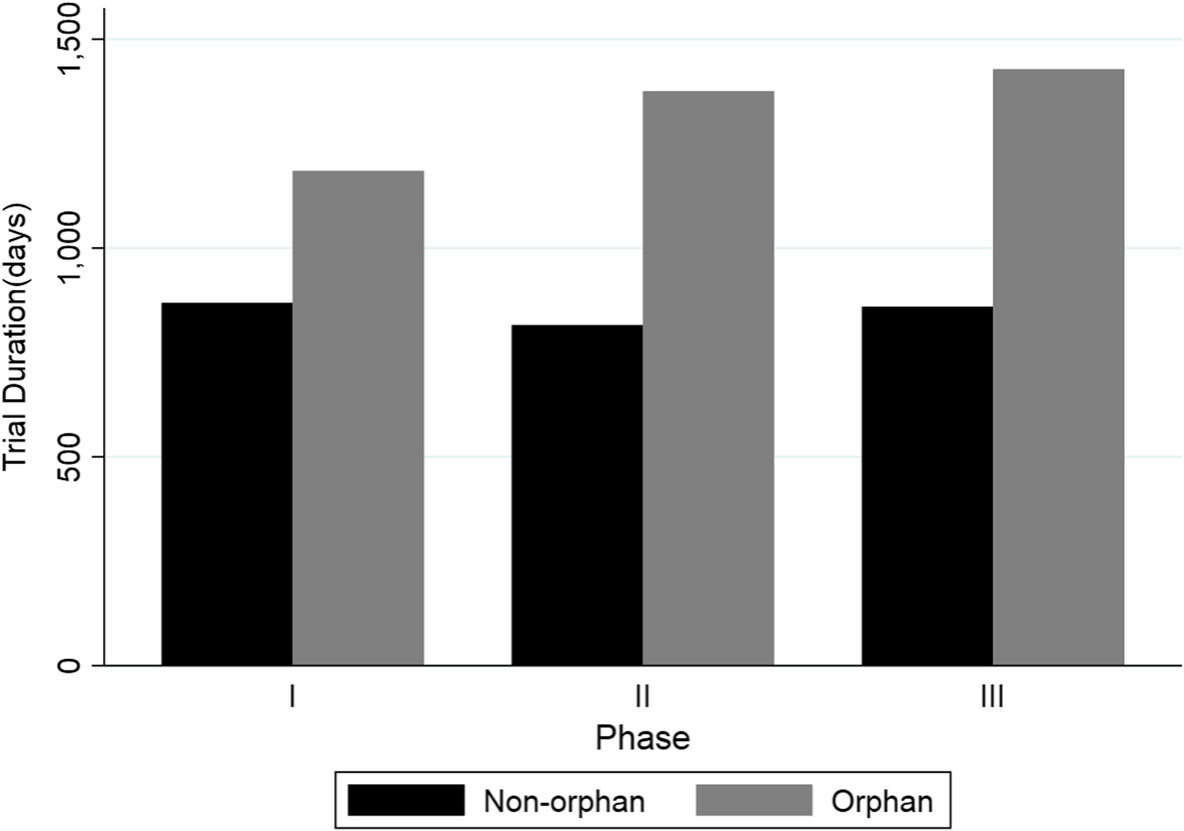
Study Duration by trial phase for NMEs only

We found that 54 out of the 100 non-orphan drug-indications were related to an approval of a NME. Out of the 100 orphan drug-indications, 74 were related to NMEs. The trial characteristics for this subgroup can be seen in Figs. 3 and 4. The resulting estimated capitalized cost per approved non-orphan NME was $489 million and the capitalized cost per approved orphan NME was $242 million (see Table 4). This resulted in the capitalized cost per approved orphan drug to be 0.50 times that of a non-orphan drug.

**Table 4:** Out-of-pocket and capitalized costs per approved drug for NMEs only

Drug Type	Phase	Expected out-of-pocket clinical costs (in millions 2013 USD)	Out-of-pocket clinical cost per approved drug (in millions 2013 USD)	Capitalized expected clinical costs (in millions 2013 USD)	Capitalized clinical cost per approved drug (in millions 2013 USD)
Non-orphan	1	$2.81	$340.30	$5.16	$488.88
	2	$7.00		$11.11	
	3	$25.75		$34.82	
	Total	$35.56		$51.09	
Orphan	1	$4.27	$137.12	$9.61	$242.46
	2	$20.86		$39.87	
	3	$20.02		$30.35	
	Total	$45.15		$79.83	

NME = New Molecular Entity

### Sensitivity Analyses

Several sensitivity analyses were conducted and the values used for these analyses are reported in Additional file 1. Table 5 reports on the results of the sensitivity analyses. Thus, when considering the uncertainty around the various parameters that were considered in this study, the out-of-pocket costs per approved non-orphan drug ranged from $218 million to $302 million while the out-of-pocket costs per approved orphan drug ranged from $100 million to $294 million. This resulted in out-of-pocket costs per orphan drug ranging from 0.36 – 1.01x of the out-of-pocket costs per approved non-orphan drug. The capitalized costs per approved non-orphan drug ranged from $360 million to $404 million while the capitalized costs per approved orphan drug ranged from $172 million to $527 million. This resulted in capitalized costs per orphan drug ranging from 0.45 – 1.28 times that of the out-of-pocket costs per approved non-orphan drug. As expected, when per patient costs for orphan drug trials are increased (up to 3.5 times), the capitalized costs for the two groups start to converge. Moreover, when the transition probabilities (success rates) of orphan drugs are reduced, the cost of development start to exceed those of non-orphan drugs.

**Table 5:** Capitalized cost per approved non-orphan and orphan drug based on sensitivity analysis parameters

	Out-of-pocket costs per approved drug			Capitalized costs per approved drug		
Parameter	Non-orphan	Orphan	Ratio (orphan/non-orphan)	Non-Orphan	Orphan	Ratio orphan/non-orphan)
Base case	$291,505,909	$166,389,643	0.57	$412,404,245	$291,263,058	0.71
Number of trials per approved drug – restricted dataset	$217,706,869	$99,866,706	0.46	$306,399,947	$172,737,264	0.56
Average number of subjects per trial – restricted dataset	$302,053,737	$152,098,329	0.50	$425,244,968	$271,257,799	0.64
Multiplier for per patient costs for phase 2 and 3 – 1.5 x	Same as base case	$104,485,324	0.36	$412,404,245	$185,473,705	0.45
Multiplier for per patient costs for phase 2 and 3 – 3.5x	Same as base case	$228,293,962	0.78	$412,404,245	$397,052,411	0.96
Trial Duration – restricted dataset	$291,505,909	$166,389,643	0.57	$406,967,750	$266,003,355	0.65
Transition Probabilities – DiMasi et al	$255,378,992	Same as base case	0.65	$360,459,277	Same as base case	0.81
Transition Probabilities, Orphan Drugs- +20% Hay et al	Same as base case	$106,023,300	0.36	Same as base case	$182,841,707	0.44
Transition Probabilities, Orphan Drugs- -20% Hay et al	Same as base case	$294,848,393	1.01	Same as base case	$526,910,607	1.28
Discount rate – 3%	Same as base case			$326,048,291	$202,067,762	0.62
Discount rate – 7%	Same as base case			$372,104,800	$249,638,586	0.67
Excluding studies that did not report on trial phase	$277,006,845	$150,354,877	0.54	$389,798,014	$272,349,673	0.70
Categorizing phase 1/2 studies as phase 2 and phase 2/3 studies as phase 3	$286,564,341	$169,382,779	0.59	$404,141,006	$293,331,017	0.73

## Discussion

In this study, we utilized publicly available clinical trial data to determine any differences in trial characteristics between non-orphan and orphan drugs; these data were then used to calculate the out-of-pocket and capitalized clinical cost of drug development for orphan and non-orphan drugs. Our analysis shows that the out-of-pocket costs per approved orphan drug is about 60% the cost for approved non-orphan drugs. Also, the capitalized costs per approved orphan drug is about 70% of the cost for their non-orphan counterparts. When focusing on NMEs alone, the capitalized cost per approved orphan drug was half that of a non-orphan drug.

The estimates in this study are directly related to differences in trial characteristics that we also investigated in this study. Publicly available data on clinical trials for both groups showed some differences in age categories and funder type as more trials for orphan drugs included children and also included partnerships between industry, NIH and other sources as a source of funding. Given that rare diseases affect children more than non-orphan diseases, [2] this is not surprising. In addition, in a competitive funding environment, it’s likely that drug development in rare diseases relies on various funding avenues in comparison to mainly industry led trials assessing non-orphan drugs. Moreover, the orphan group had a higher proportion of phase 2 trials while the non-orphan group had the highest proportion of trials in phase 3. This implies that phase 2 trials are used as pivotal trials for orphan drugs and some orphan drugs may not even be tested in a phase 3 setting.

Our analyses also estimated that the number of subjects enrolled in trials for orphan drugs are less than that of non-orphan drugs. These findings are consistent with other publications that have assessed differences between two groups [18, 19]. In addition, our analyses also demonstrated that the trial duration for trials assessing orphan drugs are longer than that of non-orphan drugs consistently for each trial phase. Longer trial durations can stem from challenges related to information on disease prevalence and incidence, lack of data on natural disease progression, timely and adequate recruitment, geographic dispersion of eligible participants and low medical expertise in the community [20, 21].

Our estimates of out-of-pocket clinical costs are different from the costs reported by DiMasi et al. Looking specifically at clinical cost per approved new drugs, DiMasi et al estimated that the out-of-pocket cost per approved drug was $965 million and the capitalized cost was $1460 million [11]. Our analysis estimated that the out-of-pocket and capitalized costs for non-orphan drugs are $291 million USD and $412 million USD respectively, much lower than the costs estimated by DiMasi et al. According to Dimasi et al, pre-clinical costs comprise of 32% of total out-of-pocket costs and 42% of total capitalized costs [11]. Thus, it should be noted that our study only looked at clinical costs per approved drug and comparison to other estimates from literature must be made with caution. Our methodology in deriving out-of-pocket costs were different from the way that DiMasi et al derived their numbers. Our numbers were based on publicly available data while DiMasi et al used confidential surveys to estimate mean phase costs. The per patient costs that we used in our analysis included costs related to clinical trial sites only (costs related to investigator and site, institutional overhead, ethics review, recruitment costs, screening, general trial procedures, drug supply, comparator drugs, efficacy assessments, laboratory procedures, site-based data management and any site-specific contract research organization costs). These costs may not be comprehensive and it is unclear which clinical trials costs were included in the DiMasi et al analysis. In light of lack of published data in this area, one can only rely on limited data and some assumptions to arrive at a reasonable estimate. Since out-of-pocket costs are directly related to expected out-of-pocket costs and capitalized costs, our estimates for all these three types are consistently less than that of DiMasi et al. Our estimates for NMEs alone, are slightly lower than the estimates in a very recent publication by Prasad et al [12] who estimated the out-of-pocket cost of developing a cancer drug to be $648 million and capitalized cost per approved cancer drug to be $757 million with 7% discount rate (2017 USD). These authors relied on publicly available data from US Securities and Exchange Commission filings for ten companies for their analyses. Thus, the differences in sources of data among the different studies can explain this variance. Moreover, our analysis focused on estimating the difference in costs of clinical development between orphan and non-orphan groups rather than computing a more accurate estimate of drug development.

Our analysis has several limitations. First, our primary data source, clinicaltrials.gov, does not register all trials. Data elements can be missing or unavailable and data provided can be inconsistent or even inaccurate [22]. Second, we combined data from different sources to estimate the mean cost of each trial phase. In the absence of robust data specific to orphan or non-orphan groups, we have made several assumptions that could potentially impact the study results. Specifically, our estimates of per-patient costs and multiplier for orphan drug per-patient costs are limited by the number of data sources available. This has large implications on the results that we have obtained. As seen in the sensitivity analysis, some data parameters heavily influence the results of this analysis. Our assumption that the per patient costs for phase 1 trials are the same for both orphan and non-orphan drugs may not hold true in cases where the treatment is toxic or where the disease is serious. This may affect our results and increase the clinical costs associated with orphan drugs. The transition probabilities and overall probability of success of orphan drugs were only available through one source (Hay et al). We conducted sensitivity analyses around this parameter (+/- 20%). With lower transition probabilities for orphan drugs, the out-of-pocket costs were similar for the orphan and non-orphan groups and capitalized costs were higher for the orphan group compared to non-orphan group. With higher transition probabilities (i.e. higher success) for orphan drugs, the out-of-pocket and capitalized costs were much lower for the orphan group. For the sake of simplicity, we did not stratify trials by country. Even if we had, per patients costs for trial subjects were only available for the United States. Morever, our dataset included data on completed trials and therefore we had to assume that trial characteristics (specifically number enrolled and trial duration) would be representative of failed trials. Finally, we did not consider the financial impact of FDA orphan drug credits and its’ effect on the out-of-pocket cost born by pharmaceutical companies. Since up to 50% of clinical trials costs for orphan drugs can be credited to the company, the estimated costs for orphan drugs portrayed here could be an overestimate. Our results indicate that, on average, the time between orphan drug designation to approval is 1665 days (4.6 years). The average length of clinical development for orphan drugs, according to our study, is 12.3 years. Thus, pivotal trial costs (phase 2 and 3) could likely be eligible for tax credits in the United States under the Orphan Drug Act. Lastly, our search strategy was not systematic meaning that we cannot be certain that relevant literature might not have been overlooked. Instead we employed a targeted approach (details available upon request). We are confident that this approach has identified key studies of interest which provide a credible evidence base on which to base conclusions.

Until now, no empirical studies have attempted to understand the differences in clinical costs of drug development for these two groups of drugs. The differences in costs that are estimated here cannot be attributed to a single factor. Rather, it’s an interplay of differences in trial size, per-patient cost by trial, clinical success rates and trial durations. It’s important to note that even with an array of sensitivity analyses, the costs for the orphan group have been consistently less than that of the non-orphan group for majority of scenarios. Our study has only focused on costs associated with clinical development. There are many other costs that are outside of clinical trials which can considered as part of drug development. These include pre-clinical costs, translational costs (from academic centres to industry), establishment of production methods and facilities to produce drug product, regulatory obligations and other out-of-pocket costs related to compassionate care programs [20]. Further studies are needed to better understand whether any of these cost categories would systematically vary between the orphan and the non-orphan groups to better understand the overall differences in drug development.

The high prices of orphan drugs are most often attributed to high cost of drug development and recouping these costs by a smaller patient pool. Without fully understanding the cost of orphan drug development, we cannot engage in any cost-based pricing approaches. Although we have shown the differences in cost for the clinical development of orphan versus non-orphan drugs, many questions still remain regarding cost-based pricing. Orphan drugs with a relatively low costs of development may still require very high prices to recoup those costs if the patient population is small enough. Even if more precise and drug-level cost of drug development becomes available, there is still a question of how much profit should be allowed beyond the cost of drug development. Much more work is still needed in this area to better understand how to reward innovation while maintaining drug budgets.

## Conclusions

Our analysis confirms that there are differences in characteristics of trials assessing orphan drugs versus non-orphan drugs including trial size and duration. We have estimated that the out-of-pocket clinical costs per approved orphan drug is $166 million compared to $291 million for non-orphan group. We estimated the capitalized clinical cost per approved orphan drug and non-orphan drug to be $291 million and $412 million respectively thus leading to a ratio of 0.71. When focusing on NMEs alone, we found that the capitalized clinical cost per approved orphan drug was half that of a non-orphan drug. Although these estimates themselves are highly dependent on the data parameters used in this analysis, our finding that orphan drug development is less than that of non-orphan drugs remain even with varied data parameters. Further research is required to better quantify the overall costs of drug development and obtain consensus on what cost categories should be included in such an analysis. Moreover, when considering value of drugs, more discussion is required before assessing whether recouping R&D costs should be a consideration when setting prices for drugs.

## Additional files

Additional file 1: Detailed Methods. (DOCX 56 kb)

Additional file 2: Mean number of subjects and study duration by trial phase. (DOCX 38 kb)

## Abbreviations

CT: Computed Tomography
FDA: Food and Drug Administration
MRI: Magnetic Resonance Imaging
NDA: New Drug Application
NIH: National Institutes of Health
NME: New Molecular Entity
R&D: Research and Development
USD: US Dollars
