# Supplementary material for: Estimating the clinical cost of drug development for orphan versus non-orphan drugs
Source: Orphanet J Rare Dis. 2019 Jan 10;14:12. doi: 10.1186/s13023-018-0990-4 (PMC6327525; doi:10.1186/s13023-018-0990-4)
Supplement: Supplementary file 1 — Detailed Methods. (DOCX 56 kb) [file 13023_2018_990_MOESM1_ESM.docx]

**Additional file 1: Detailed Methods**

Using the FDA Orphan Drug Database (https://www.accessdata.fda.gov/scripts/opdlisting/oopd/), we identified all orphan drugs that have been approved from January 2000 to December 2015. The orphan drugs were sorted randomly and the top 100 drugs were selected for the purpose of this analysis.

In order to obtain a list of non-orphan drugs, use the Drugs@FDA database (<http://www.accessdata.fda.gov/scripts/cder/daf/>) and download all approvals from January 2000 to December 2015. Approvals related to orphan drugs, new formulations, new manufacturers, new dosage forms and over the counter switches were eliminated from this list. This list was sorted randomly, and the first 100 drugs were selected for the analysis to constitute the “non-orphan” drugs.

Table 1: Variable names and descriptions derived from the datasets under analysis

| Variable description | Variable name | |
| --- | --- | --- |
|  | Non-orphan | Orphan |
| Mean number of trials by phase per approved drug | a_d,i,_ | a_o,i_ |
| Mean number of subjects enrolled by phase | n_d,i_ | n_o,i_ |
| Mean trial duration by phase | t_d,i_ | t_o,i_ |

Where i = 1,2 or 3 representing phase 1, 2 and 3 respectively.

Based on the variables above, we then calculated the average cost per trial phase for non-orphan drugs, c_d,i_, using the following formulas:

c_d,i_ = a_d,i_ x n_d,i_ x b_d,i_

Similarly, the average cost per trial phase for orphan drugs was calculated by:

c_o,i_ = a_o,i_ x n_o,i_ x b_o,i_

The variables b_d,i_ and b_o,i_ represents the per patient cost for non-orphan drugs for phase i and the per patient cost for orphan drugs for phase i. Our dataset did not have information on b_c,i_ and b_o,i_. Therefore, we relied on external sources for this information. Biopharmaceutical Industry-Sponsored Clinical Trials: Impact on State Economies’ report contained the following information in 2013 USD for all drugs [1].

| Phase | Average per patient Clinical trial costs, USD 2013 |
| --- | --- |
| 1 | 38,500 |
| 2 | 40,000 |
| 3 | 42,000 |

Another report by EvaluatePharma has recently reported on the average cost of phase 3 trials and corresponding average patient numbers for common and rare diseases [2]. Based on this data, the cost per patient for phase 3 clinical trials was calculated to be 2.5x higher for orphan drugs versus non-orphan drugs. Given the lack of further data in this area, we used 2.5 multiplier for phase 2 and 3 and the cost per patient for phase 1 was assumed to be the same between the two groups.

| Variable | Value (2013 USD) |
| --- | --- |
| b_c,1_ | 38,500 |
| b_c,2_ | 40,000 |
| b_c,3_ | 42,000 |
| b_o,1_ | 38,500 |
| b_o,2_ | 100,000 |
| b_o,3_ | 105,000 |

Once the c_d,i_ and c_o,i_ were calculated for each phase, we then calculated the expected cost for each trial phase for each non-orphan and orphan groups:

E(c_d,1_) = c_d,1_

E(c_d,2_) = s_d,1-2 x_ c_d,2_

E(c_d,3_) = s_d,1-2 x_ s_d,2-3 x_ c_d,2_

Where s_d,1-2_ and s_d,2-3_ represents respectively the probability of success for moving from phase 1 to 2 and 2 to 3 for non-orphan drugs. These transition probabilities take into account the successful and unsuccessful trials at each phase.

DiMasi et al includes a similar calculation when calculating the cost of drug development per approved drug. Our dataset only includes information on successful trials. When calculating this expected costs, we have made the assumption that the trial characteristics (number of trials by phase, number of subjects involved and trial duration) are the same for successful and unsuccessful trials.

The data on transition probabilities were derived from Hay et al [3]. Their work included transition probabilities for all drugs and orphan drugs separately. For the purpose of our analysis, we have included the transition probabilities for all drugs as reflecting non-orphan drugs:

| Variable | Hay et al, 2014 [3] | DiMasi et al, 2016 [4] |
| --- | --- | --- |
| s_d,1-2_ | 64.5% | 59.52% |
| s_d,2-3_ | 32.4% | 35.52% |
| s_d,3-f_ | 60.1% | 61.95% |
| s_d,f-a_ | 83.2% | 90.35% |
| s_o,1-2_ | 86.8% |  |
| s_o,2-3_ | 70.0% |  |
| s_o,3-f_ | 66.9% |  |
| s_o,f-a_ | 81.0% |  |

Variables s_d,3-f_ and s_d,f-a_ represent the probability of success of going from phase 3 to regulatory filing and regulatory filing to approval respectively. The transition probabilities for all drugs from Hay et al are similar to the DiMasi et al 2016 transition probabilities.

In order to determine the out-of-pocket clinical period cost per approved new drug, we used the following formula:

Out-of-pocket clinical period cost per approved drug = [E(c_d,1_) + E(c_d,2_) + E(c_d,3_)]/[ s_d,1-2_ x s_d,2-3_ x s_d,3-f_ x s_d,f-a_]

In order to include the opportunity cost of funds invested in new chemical entity research and development for a full cost estimate, we then capitalized the mean phase costs to the point of marketing approval.

The total length of time (in days) from phase 1 to regulatory approval, T, was calculated as follows for the non-orphan and orphan groups:

T_d_ = t_d,1_ + t_d,2_ + t_d,3_ + t_d,f-a_

T_o_ = t_o,1_ + t_o,2_ + t_o,3_ + t_o,f-a_

The values for t_d,1_, t_d,2_, t_d,3_, t_o,1_, t_o,2_ and t_o,3_ were calculated based on this dataset. The variable t_c,f-a_ represents time from filing to approval. The average time for FDA standard review was obtained from the FDA (12 months). Since some orphan drugs may be eligible for priority review (~ 6 months) we took a sample of our orphan drug dataset to see the proportion approved via priority review. It was found that ~60% of OD reviews were priority and therefore we applied a weighted approval time of 10 months for orphan drugs.

Using an interest rate of 10.5% per annum as per the methods in DiMasi et al [4], we then calculated the capitalized mean phase costs:

c_d,1,c_= c_d,1_ [ 1 + [(T_d_)*(0.105/365)]]

c_d,2,c_ = c_d,2_ [ 1 + [(T_d_ – t_d,1_)*(0.105/365)]]

c_d,3,c_ = c_d,3_ [ 1 + [(T_d_ – t_d,1_ – t_d,2_)*(0.105/365)]]

Similarly, the capitalized mean phase costs for the orphan group was also calculated.

For each group, the capitalized expected phase costs were then calculated.

E(c_d,1,c_) = c_d,1,c_

E(c_d,2,c_) = s_d,1-2_ x c_d,2,c_

E(c_d,3,c_) = s_d,1-2_ x s_d,2-3_ x c_d,3,c_

The capitalized clinical period cost per approved new drug can be estimated by:

Capitalized clinical period cost per approved non-orphan drug = [E(c_d,1,c_) + E(c_d,2,c_) + E(c_d,3,c_)]/[ s_d,1-2_ x s_d,2-3_ x s_d,3-f_ x s_d,f-a_]

Capitalized clinical period cost per approved orphan drug = [E(c_o,1,c_) + E(c_o,2,c_) + E(c_o,3,c_)]/[ s_o,1-2_ x s_o,2-3_ x s_o,3-f_ x s_o,f-a_]

The variability in results were assessed with sensitivity analysis around many of the data parameters as seen in the table below.

Table: Inputs for sensitivity analyses

**References:**

1. Practice, B.T.P., Biopharmaceutical Industry-Sponsored Clinical Trials: Impact on State Economies. 2015.

2. EvaluatePharma, Orphan Drug Report 2015. 2015.

3. Hay, M., et al., Clinical development success rates for investigational drugs. Nat Biotechnol, 2014. 32(1): p. 40-51.

4. DiMasi, J.A., H.G. Grabowski, and R.W. Hansen, Innovation in the pharmaceutical industry: New estimates of R&D costs. J Health Econ, 2016. 47: p. 20-33.

5. Prasad, V. and S. Mailankody, Research and Development Spending to Bring a Single Cancer Drug to Market and Revenues After Approval. JAMA Intern Med, 2017. 177(11): p. 1569-1575.

6. Light, D.W. and R. Warburton, Demythologizing the high costs of pharmaceutical research. BioSocieties, 2011. 6(1): p. 34-50.
