# Supplementary material for: Estimating the clinical cost of drug development for orphan versus non-orphan drugs
Source: Orphanet J Rare Dis. 2019 Jan 10;14:12. doi: 10.1186/s13023-018-0990-4 (PMC6327525; doi:10.1186/s13023-018-0990-4)
Supplement: Supplementary file 2 — Mean number of subjects and study duration by trial phase. (DOCX 38 kb) [file 13023_2018_990_MOESM2_ESM.docx]

**Additional file 2: Mean number of subjects and study duration by trial phase**

Table A: Mean number of subjects in each trials phase for non-orphan and orphan groups

Table B: Study Duration for each trial phase for non-orphan and orphan groups
